# Do obese but metabolically normal women differ in intra-abdominal fat and physical activity levels from those with the expected metabolic abnormalities? A cross-sectional study

**DOI:** 10.1186/1471-2458-10-723

**Published:** 2010-11-24

**Authors:** Louise Hayes, Mark S Pearce, Michael J Firbank, Mark Walker, Roy Taylor, Nigel C Unwin

**Affiliations:** 1 Institute of Health and Society, Newcastle University, Newcastle-upon-Tyne, UK; 2 Institute for Ageing and Health, Newcastle University, Newcastle-upon-Tyne, UK; 3 Institute of Cellular Medicine, Newcastle University, Newcastle-upon-Tyne, UK; 4 Faculty of Medical Sciences, University of the West Indies, Bridgetown, Barbados

## Abstract

**Background:**

Obesity remains a major public health problem, associated with a cluster of metabolic abnormalities. However, individuals exist who are very obese but have normal metabolic parameters. The aim of this study was to determine to what extent differences in metabolic health in very obese women are explained by differences in body fat distribution, insulin resistance and level of physical activity.

**Methods:**

This was a cross-sectional pilot study of 39 obese women (age: 28-64 yrs, BMI: 31-67 kg/m^2^) recruited from community settings. Women were defined as 'metabolically normal' on the basis of blood glucose, lipids and blood pressure. Magnetic Resonance Imaging was used to determine body fat distribution. Detailed lifestyle and metabolic profiles of participants were obtained.

**Results:**

Women with a healthy metabolic profile had lower intra-abdominal fat volume (geometric mean 4.78 l [95% CIs 3.99-5.73] vs 6.96 l [5.82-8.32]) and less insulin resistance (HOMA 3.41 [2.62-4.44] vs 6.67 [5.02-8.86]) than those with an abnormality. The groups did not differ in abdominal subcutaneous fat volume (19.6 l [16.9-22.7] vs 20.6 [17.6-23.9]). A higher proportion of those with a healthy compared to a less healthy metabolic profile met current physical activity guidelines (70% [95% CIs 55.8-84.2] vs 25% [11.6-38.4]). Intra-abdominal fat, insulin resistance and physical activity make independent contributions to metabolic status in very obese women, but explain only around a third of the variance.

**Conclusion:**

A sub-group of women exists who are metabolically normal despite being very obese. Differences in fat distribution, insulin resistance, and physical activity level are associated with metabolic differences in these women, but account only partially for these differences. Future work should focus on strategies to identify those obese individuals most at risk of the negative metabolic consequences of obesity and on identifying other factors that contribute to metabolic status in obese individuals.

## Background

The health consequences of obesity are well established. Obese individuals are at greater risk of cardiovascular disease[1,2], largely through the association of obesity with dyslipidaemia, hypertension, glucose intolerance and diabetes[3]. It is well known that individuals who are not obese can develop these metabolic abnormalities[4,5]. What has been less widely appreciated until recently is that a subset of obese individuals remains free of any of these metabolic abnormalities. Current data suggest that 20-30% of obese individuals appear to maintain a favourable metabolic profile, more usually associated with lean individuals[6,7]. Such individuals have been termed 'metabolically normal obese'[8], 'metabolically healthy but obese'[9], 'obese metabolically normal'[10] or described as having metabolically benign obesity[11] or uncomplicated obesity[12]

The mechanisms through which obesity exerts its effect on metabolic disorders remain to be fully explained, but considerable interest has focused on the role of body fat distribution. Vague first identified android (upper-body or abdominal) obesity as being associated with diabetes and atherosclerosis over 50 years ago [13]. Individuals with higher levels of central adiposity are at greater risk of developing lipid and glucose abnormalities than those with fat distributed more peripherally [14,15]. Compelling evidence now exists to suggest that excess intra-abdominal (visceral) fat in particular confers an increased risk of developing a metabolic disorder[16,17].

An increasing body of work exists to suggest that poor metabolic health associated with obesity is mediated by physical activity. Several studies have demonstrated that higher physical activity and/or cardiorespiratory fitness is associated with more favourable metabolic health even among obese individuals[7,18-22].

Several recent studies have identified factors that are associated with high insulin sensitivity and a favourable metabolic profile in obese individuals. Although standard criteria to define the metabolically healthy obese (MHO) individual are lacking, studies have suggested that both lower intra-abdominal fat (IAF)[8,23] and higher physical activity level[24] are associated with a more healthy metabolic profile in obese individuals.

However, data on the association of directly measured IAF and other cardiovascular risk factors with metabolic health in the very obese (BMI ≥ 35) are scarce. Most work in this field has focused on the overweight and modestly obese (BMI 25-34)[11,25].

This paper presents a pilot study that arose from the clinical observation that a substantial minority of individuals referred to a metabolic clinic for the management of severe obesity had a healthy metabolic profile. Our aim was to determine to what extent differences in metabolic health in very obese (class II and class III obesity) women are explained by differences in body fat distribution, insulin resistance and level of physical activity.

## Methods

### Participants

Participants in this study were 39 very obese women (median BMI 39.6 kg/m^2^), aged 28-64 years and of Caucasian origin. The women were recruited from a number of sources, including an obesity clinic, dieticians and from responding to a press release. Participants were defined as 'metabolically healthy' (MHO) if they had a fasting plasma glucose less than 6.1 mmol/l^-1^, a 2-hour post 75 g glucose challenge plasma glucose less than 7.8 mmol/l^-1^, fasting triglycerides less than 2.2 mmol/l^-1^, HDL cholesterol greater than 1.1 mmol/l^-1^and blood pressure < 160/95 mmHg (n = 20). Participants not meeting these criteria were defined as 'metabolically abnormal' (MetAb). At the time this study was planned there was a lack of consensus on appropriate cut points for metabolic abnormalities, and for example, no internationally agreed definition of the metabolic syndrome. Our approach to defining MHO was therefore somewhat pragmatic, combining WHO cut points for glucose with cut points recommended locally and used in care for people with diabetes and treatment within the obesity service for lipids and blood pressure. Nineteen women (9 MHO and 10 MetAb) were postmenopausal; four (1 MHO; 3 MetAb) were currently using HRT.

Ethical approval for the study was obtained from the Joint Ethics Committee of Newcastle and North Tyneside Health Authority and the Newcastle Universities. Participants provided written consent for their participation.

### Assessment of body fat distribution

Body fat was assessed using magnetic resonance imaging (MRI). Images were acquired on a Philips Intera 1.5 T MRI system and rapid T1-weighted spin-echo sequence with repetition time = 36 ms, echo time = 14 ms, flip angle 120°, field of view = 60 cm, 256 × 256 matrix and phase-conjugate symmetry. Participants were scanned from the lower sternum to the mid thigh, by acquiring 10 mm thick transverse images with 30 mm gaps between slices, as they lay in a supine position.

The volume of IAF and SCF fat compartments were determined based on the method of Thomas[26]. The MR images were transferred from the scanner to a PC for analysis with custom written software. Fat appears as a bright, high-intensity signal against a lower signal from other tissues. Total fat content was determined in the following fashion for each slice in the image. To automatically locate the outside of the torso, the software examines pixels starting at the posterior left of the image, and moving diagonally inwards, until two adjacent pixels brighter than 1/3 of the maximum signal intensity are found. A threshold value is determined from the sharpest edge in a 5 × 5 pixel neighbourhood of this point, and a contour around the torso automatically generated using this threshold. The mean (M_c_) and standard deviation (SD_c_) of the pixels along this contour are determined and a final threshold T_f _is set as T_f _= M_c _- SD_c_.

A new contour around the outside of the torso is automatically generated using this threshold, and those pixels with an intensity brighter than T_f _are designated as fat. The images were manually checked, and the contour, and/or threshold level could be adjusted as necessary. Each slice was reviewed manually to delete unwanted voxels associated with, for example, bone marrow and some stomach contents which give high intensity signals in the images. The total fat volume was then determined by multiplying the number of pixels brighter than the threshold T_f _by the pixel size and sum of slice thickness and slice gap.

Abdominal adipose tissue was defined as fat found in the slices from the femoral heads to the top of the liver or base of the lungs. Subcutaneous fat in this region was identified as SCF. Fat that was within the peritoneum was identified as IAF. To determine the IAF content, a region encompassing the viscera was drawn manually by an operator using a computer mouse. The IAF volume was determined by multiplying the number of pixels brighter than the threshold T_f _in this region by the pixel size and sum of slice thickness and slice gap. The subcutaneous fat volume was determined by subtracting the intra-abdominal fat volume from the total fat volume. A measure of thigh fat was made by calculating the fat area in one slice of one leg taken 80 mm (2 slices) below the femoral heads slice.

### Anthropometric measurements

Height was measured without shoes to the nearest 5 mm, with the head positioned so that the eye and the external auditory meatus were level. Weight was measured to the nearest 100 g with the subject lightly clothed. Waist circumference was measured, with the waist unclothed and hip circumference over the subject's underwear, to the nearest centimetre at the midpoint between the lower costal margin and the superior iliac crest, and the hips over the greater trochanters. Sagittal diameter was measured using an abdominal caliper as the distance between the highest point of the abdomen and the examination table with the participant lying supine. Body Mass Index (BMI) was calculated as weight (kg)/[height(m)^2^].

### Biochemical measurements

Participants attended for screening after an overnight fast. Participants followed their usual diet and physical activity pattern prior to screening. Fasting blood samples were taken on arrival at the clinic. Participants then took a standard oral glucose tolerance test. A venous blood was taken after 30 minutes and 2 hours. Plasma was separated by centrifugation within an hour (after refrigeration of the sample). Serum insulin levels were measured by an enzyme linked immunosorbent assay (ELISA; Dako Ltd, Ely, UK). Plasma glucose concentrations were measured on a Yellow Springs Analyser (YSI Stat Plus 2300; Yellow Springs Instruments, Farnborough, UK). Insulin resistance (HOMA-IR) was estimated using the Homeostasis Model Assessment, based on fasting insulin and glucose[27].

Serum triglycerides were measured by a lipase/glycerol kinase method on a Cobas Bio centrifugal analyser (Roche Products Ltd, Welwyn, UK) using a commercial kit (Sigma Diagnostics, Poole, UK). Serum HDL cholesterol was estimated by measuring the supernatant cholesterol concentration after precipitation of apolipoprotein B containing lipoproteins with heparin and manganese. LDL cholesterol was estimated using the Friedwald formula. Non-esterfied fatty acid (NEFA) was measured by a WAKO cabas analyser.

### Blood pressure

Blood pressure was measured twice in the right arm using an automated blood pressure monitor (Omron M5-I) and a large cuff (for arm circumference 32-42 cm). The mean measurement was used in analysis.

### Physical activity

Current level of physical activity was assessed by an interviewer-administered questionnaire, based on the Modifiable Activity Questionnaire[28], and by the use of a pedometer (Yamax Digiwalker SW-200). The Modifiable Activity Questionnaire collects information on leisure time, occupational and transportation (walking or cycling) physical activity. The amount of leisure time, occupational and transportation physical activity undertaken was recorded and assigned a metabolic equivalent score (MET; 1 MET = 3.5 ml O_2 _uptake per kg/min) according to the Compendium of Physical Activities[29]. The reported daily average time spent in leisure time activities, occupational activities (including sitting) and walking or cycling for transport was summed and total energy expenditure was computed assuming that time not reported (i.e. 24 hours minus the sum of the hours reported) was spent at rest and assigned a MET score of 1. In addition participants were categorised according to whether or not they met the current UK recommendation for the level of physical activity required to provide health benefits (at least 30 minutes of moderate activity on at least 5 occasions per week[30]).

Participants wore the pedometer for four days (1 weekend day and 3 weekdays). A daily mean number of steps walked was calculated.

### Statistical analyses

All data analyses were performed using SPSS version 11.0. Measures of central tendency and variability are presented for anthropometric and biochemical data. Variables were log transformed where appropriate and geometric means with 95% confidence intervals are presented. Where variable distributions were not normalised by log transformation medians and inter-quartile ranges are presented. Independent samples *t*-tests or Mann-Whitney tests were used to compare means or medians between cases and controls as appropriate. Pearson correlations were calculated to evaluate the relationship between IAF and SCF and glucose, lipids and blood pressure. Multivariable logistic regression was used to identify determinants of MHO status. Odds ratios (OR) and corresponding 95% confidence intervals (CIs) are reported. The amount of variance explained by independent variables in logistic regression analysis was calculated as the difference in deviance between the null model and the model with the independent variable divided by the deviance in the null model[31]. A *p *value < 0.05 was considered statistically significant.

## Results

### Body fat distribution and metabolic status

**Figure 1:**
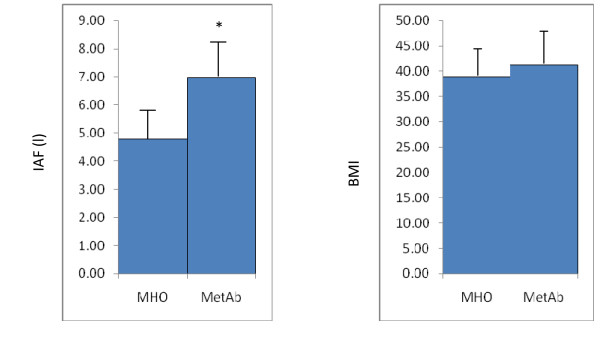
**Comparison of MRI determined intra-abdominal fat (IAF) and BMI in metabolically normal (MHO n = 20) and those with any metabolic abnormality (MetAb n = 19)**. Geometric mean presented for IAF; median IQR for BMI. The symbol * indicates the groups are significantly different from each other (p < 0.01)

Twenty participants were classified as MHO, the remaining 19 as MetAb. Participant characteristics are shown in table 1. The MHO group differed significantly from the MetAb group on each of the criteria upon which participants were defined as MHO or MetAb except systolic blood pressure (p = 0.193). A fairly large (2.4 kg/m^2^) non-significant difference in BMI between the MHO and MetAb groups was found (Figure 1). Analyses were repeated with adjustment for BMI. This did not affect the results of the analyses and the unadjusted results are presented here. The MHO group had significantly less IAF than the MetAb (4.8 l vs 6.9 l; p = 0.006). The groups did not differ significantly in age.

**Table 1:** Participant characteristics

	MHO		MetAb		
**n**	**20**		**19**		**p**

	**Central tendency**	**Variability**	**Central tendency**	**Variability**	

Fasting glucose (mmol/l^-1^)^a^	5.37	(5.19-5.69)	6.17	(5.75-7.6)	< 0.001
2-hour glucose (mmol/l^-1^)^b^	5.63	(5.13-6.18)	7.72	(6.71-8.89)	0.001
Triglycerides (mmol/l^-1^)^c^	1.40	(0.43)	2.34	(0.72)	< 0.001
HDL cholesterol (mmol/l^-1^)	1.48	(0.24)	1.32	(0.24)	0.036
Systolic BP (mmHg)^b^	119.5	(113.3-126.0)	125.6	(118.4-135.4)	0.193
DiastolicBP (mmHg)^b^	85.1	(82.5-87.8)	91.7	(86.4-97.4)	0.037
					
Weight (kg)^a^	99.5	(88.2-112.3)	108.9	(93.7-119.5)	0.213
Body Mass Index (kg/m^2^)^a^	38.9	(35.1-43.7)	41.3	(38.1-47.9)	0.137
Age (yrs) ^c^	46.1	(9.8)	47.5	(9.6)	0.651

^a ^Median (interquartile range), Mann-Whitney test for difference; ^b ^Geometric mean (95% confidence intervals); ^c ^Mean (SD)

**Figure 2:**
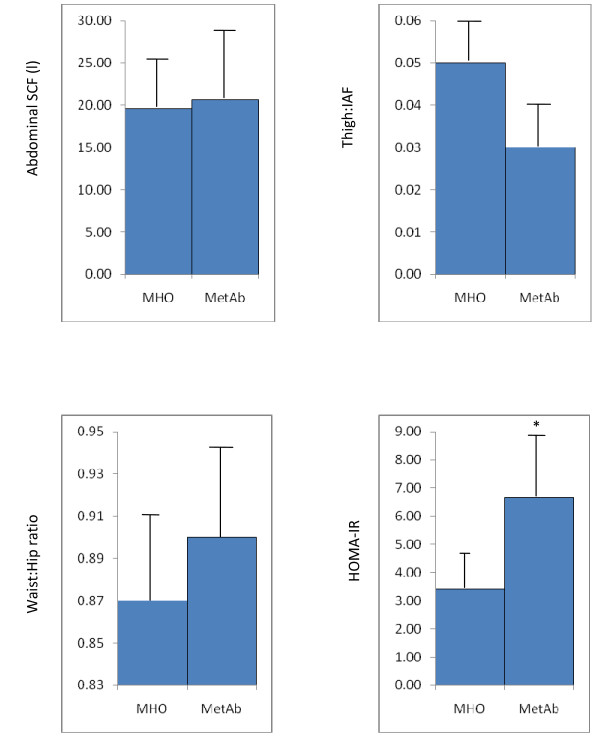
**Comparison of abdominal subcutaneous fat (SCF), thigh fat:IAF, waist:hip ratio (WHR) and HOMA-IR in metabolically normal (MHO n = 20) and those with any metabolic abnormality (MetAb n = 19**. All values are geometric means with 95% CI, except WHR (mean; 95% CI). The symbol * indicates the groups are significantly different from each other (p < 0.01)

The MHO group had significantly lower insulin resistance, as estimated by HOMA (3.41 vs 6.67; p = 0.002) (Figure 2). The 2 groups did not differ significantly in abdominal SCF, thigh fat:IAF or waist:hip ratio (WHR).

Correlations between IAF and SCF and glucose, lipids, blood pressure and HOMA-IR are shown in table 2. IAF and SCF were significantly correlated with each other (r = 0.57, p < 0.001). IAF, but not SCF was significantly and positively correlated with postload glucose (r = 0.31) and systolic blood pressure (r = 0.35) and negatively with HDL (r = -0.34). The correlation between IAF and triglycerides approached statistical significance (r = 0.31, p = 0.054), but there was no evidence of a correlation between SCF and triglycerides. Both IAF and SCF were significantly correlated with diastolic blood pressure (r = 0.31 and r = 0.41 for IAF and SCF respectively). HOMA estimated insulin resistance was not significantly correlated with either IAF or SCF.

**Table 2:** Correlations of fat volumes with fasting and postload glucose, triglycerides, HDL, blood pressure and HOMA-IR

	IAF	SCF
	**r**	**r**

Fasting glucose (mmol/l^-1^)	0.21	0.15
2-hour glucose (mmol/l^-1^)	0.31^†^	0.08
Triglycerides (mmol/l^-1^)	0.31	0.08
HDL cholesterol (mmol/l^-1^)	-0.34^†^	-0.02
SBP (mmHg)	0.35^†^	0.24
DBP (mmHg)	0.31^†^	0.41^†^
HOMA-IR	0.25	0.23

^† ^p < 0.05

### Physical activity

Physical activity questionnaire data and pedometer data are summarised in table 3. The MHO group recorded a higher mean number of steps walked per day (6548 vs 5427 in the MetAbs) but this difference was not statistically significant (p = 0.252) and the inclusion of pedometer recorded steps per day in the logistic regression model did not contribute to the explanatory power of the model. MHOs reported greater daily energy expenditure in leisure time activities than the MetAbs, both over the longer term (past year) and in the preceding week, but these differences were not statistically significant. Total energy expenditure for the 2 groups was very similar for past year (slightly higher in MHOs) and past week (slightly higher in MetAbs).

**Table 3:** Self-reported and pedometer recorded physical activity

	MHO		MetAb		
**n**	**20**		**19**		**p**

	Mean	(SD)/[CI]	Mean	(SD)/[CI]	
Steps per day (pedometer)^a^	6548.2	(2997.8)	5427.1	(2938.2)	0.252
DEE Past year leisure (Kcal)^b^	236.4	[155.5-359.5]	167.5	[111.2-252.2]	0.332
DEE Past week leisure (Kcal)^b^	239.2	[157.1-364.3]	151.9	[89.5-257.5]	0.244
DEE Past year occupational (Kcal)^b^	484.9	[355.3-661.9]	508.2	[358.5-720.4]	0.851
DEE Past week occupational (Kcal)^b^	513.3	[375.3-701.9]	498.3	[347.7-714.0]	0.912
Total DEE Past year (Kcal)^a^	2687.5	(810.5)	2590.2	(684.5)	0.684
Total DEE Past week (Kcal)^a^	2657.7	(818.4)	2711.7	(762.1)	0.830
					
	%	[95% CI]	%	[95% CI]	
Moderate activity × 5 per week	70.0	[55.8-84.2]	25.0	[11.6-38.4]	0.010

DEE = Daily Energy Expenditure; ^a ^Mean (SD), independent samples t-test for difference between MENO and MetAB; ^b ^Geometric mean (95% confidence intervals [CIs])

Although the groups did not differ significantly in terms of their self-reported total energy expenditure there was a significant difference between the groups in the proportion of participants reporting meeting current guidelines for the level of physical activity required to have an impact on health. Seventy per cent of the MHO group reported participating in at least 30 minutes of moderate physical activity on at least 5 occasions per week (the current UK Government guideline [30]), compared to 25% of the MetAb group (p = 0.010).

### Contribution of body fat distribution and physical activity to metabolic status

In logistic regression analysis, a greater volume of IAF was associated with higher risk of having a metabolic abnormality (OR per litre increase in IAF = 1.48, 95% CIs 1.07-2.05, p = 0.018). Subcutaneous abdominal fat, thigh fat and total abdominal fat were not associated with MHO status. Controlling for the effect of SCF, SCF and BMI or SCF, BMI and waist circumference did not affect the relationship between IAF volume and MHO status (table 4). HOMA-estimated insulin resistance was strongly significantly associated with an increased likelihood of being MetAb (OR = 6.66, 95% CIs 1.57-28.16, p = 0.01). When the effect of HOMA was controlled for IAF was still significantly associated with metabolic status (p = 0.046). IAF explained 14.5% of the variance in MHO status. HOMA explained an additional 15%. An interaction between IAF and HOMA was also found, (OR = 1.31, 95% CIs 1.11-1.56) which explained slightly more (32% for main effects plus interaction) of the variance than IAF and HOMA together.

**Table 4:** Logistic regression analysis to identify determinants of being MetAb

	OR	95% CIs	p
*Univariate analysis*			
IAF	1.48	(1.07-2.05)	0.018
SCF	1.02	(0.94-1.11)	0.636
All abdominal fat	1.04	(0.97-1.12)	0.296
Thigh fat	0.99	(0.93-1.06)	0.836
HOMA-IR	6.66	(1.57-28.16)	0.010
Physically active^†^	0.14	(0.04-0.58)	0.006
			
*Multivariable analysis*			
*IAF + SCF*			
IAF	1.68	(1.14-2.49)	0.009
SCF	0.92	(0.82-1.04)	0.194
			
*IAF, SCF + BMI*			
IAF	1.68	(1.13-2.48)	0.010
SCF	0.91	(0.73-1.12)	0.376
BMI	1.02	(0.86-1.20)	0.853
			
*IAF, SCF, BMI + waist*			
IAF	1.66	(1.08-2.55)	0.021
SCF	0.91	(0.73-1.13)	0.380
BMI	1.01	(0.83-1.23)	0.931
waist	1.01	(0.92-1.10)	0.911
			
*IAF + HOMA-IR*			
IAF	1.39	(1.01-1.92)	0.046
HOMA-IR	5.36	(1.23-23.41)	0.025
			
*IAF + Physically active*			
IAF	1.44	(1.02-2.03)	0.041
Physically active	0.19	(0.04-0.83)	0.028
			
*IAF + HOMA-IR + Physically active*			
IAF	1.39	(0.98-2.01)	0.077
HOMA-IR	4.68	(1.13-19.4)	0.034
Physically active	0.16	(0.03-0.96)	0.045

^† ^Defined as self-reporting being moderately active for 30 min at least 5 times per week

Meeting current UK Government guidelines for physical activity participation was negatively associated with being MetAb (OR for meeting the guidelines = 0.14, 95% CIs 0.04-0.58, p-0.006) and explained 15.2% of the variance. When IAF and physical activity were included in the same model the effect of both was attenuated only slightly. When HOMA was added to the model HOMA and physical activity remained independent predictors, together explaining 38.2% of the variance, but IAF was no longer a statistically significant predictor (p = 0.077), although it did contribute to the explanatory power of the model.

## Discussion

We have demonstrated in this study of very obese women that lower intra-abdominal fat volume, less insulin resistance and higher level of physical activity are factors that influence metabolic function beneficially. The results of multivariable modelling suggest that each makes an independent contribution to the risk of having a metabolic abnormality, suggesting that the impact of physical activity on metabolic health is independent of its effect on IAF and that IAF itself makes an independent contribution and does not only operate via insulin resistance. Our findings support a growing body of evidence that individuals exist who are very obese (in this study median BMI of 39.6) but remain metabolically normal.

However, together IAF, HOMA-IR and meeting current guidelines for participation in physical activity accounted for only a little over a third of the risk of having a glucose, lipid or blood pressure abnormality in this group of women. Much of the variance in metabolic status in these women remains unexplained by the variables considered here.

In contrast to other studies[15,32,33] we did not find an association between abdominal SCF and presence of a metabolic abnormality. Although the MHO and MetAb groups did not differ in volume of total abdominal SCF, differences may exist in deep and superficial subcutaneous fat depots, which were not examined in this study. Alternatively, in this very obese population SCF may play a less important role in the metabolic complications of adiposity than in less obese groups.

Characteristics of IAF that support its importance in terms of metabolic health, including the lower sensitivity of IAF to the anti-lipolytic effect of insulin, greater sensitivity to the lipolytic effect of catecholamines and role as a source of cytokines have been well documented [5,34].

It has also been suggested that an excess of IAF may act as a marker for an impairment in an individual's ability to store fat in the subcutaneous depot or to utilise it efficiently within mitochondria [35]. This suggests that the MHO women in this study are better able to store fat appropriately than those with a metabolic abnormality who store excess energy as IAF, and also potentially in skeletal muscle and the liver.

Insulin resistance, as estimated by HOMA, was an independent predictor of metabolic status, and explained a comparable amount of the variance to IAF. It should be noted, however, that it was to be expected that the MHO women would have lower HOMA values as normal glycaemic control, a component of HOMA, was part of the definition of MHO.

It was surprising that neither IAF nor SCF were significantly correlated with insulin resistance in this study. This could be attributed to the use of HOMA-IR as a proxy for insulin resistance in this study. Although HOMA-IR and the euglycaemic hyperinsulinaemic glucose clamp procedure have a good correlation in larger studies (0.6-0.88)[36] HOMA-IR may have less utility in a small study. Furthermore, HOMA-IR is closely correlated with fasting insulin and other investigators have reported no association or a weak association between fasting insulin and/or glucose and IAF[37,38] even when a significant relationship between glucose disposal and IAF exists[37].

The participants in this study, and especially those with normal metabolic function, reported higher levels of physical activity than have been reported in other studies, including, for example, the Health Survey for England 2008, which reported only 19% of obese women meet the current guideline[39]. The difficulties associated with collecting data on physical activity level have long been acknowledged[40]. However, there is no reason to believe that the MHO and MetAb groups would have reported their physical activity habits systematically differently, and as the focus of the study was on differences between the two groups, this does not present a major threat to the validity of these data. Nevertheless it would be desirable in future work to have a more sophisticated measure of physical activity using an accelerometer and/or heart rate monitoring to provide more precise information on how energy expenditure and physical activity duration and intensity differ between those who maintain normal metabolic function and those who develop an abnormality.

Our results could have important public health and clinical significance. Despite widespread awareness of the negative consequences of excess adiposity and efforts to redress or at least halt the rise in obesity, its prevalence continues to increase. Not all obese individuals have the glucose, lipid and blood pressure abnormalities usually associated with obesity. This suggests that there is a need for a different approach to the problem, which focuses on those most at risk of developing the ill health consequences of obesity and who should be targeted for intervention. The findings of this pilot study suggest that IAF accumulation, insulin resistance and lack of physical activity each have an independent role to play in identifying those most at risk of the negative metabolic health consequences of obesity. Future work should focus on strategies to identify individuals most likely to benefit from intervention, for example, by encouraging the routine use of established simple methods, including the measurement of waist circumference and the use of dual energy X-ray absorptiometry (DXA) where available, to identify those likely to have high levels of IAF. In addition work is needed to identify factors that drive deposition of fat in the visceral depot. Many determinants of IAF accumulation have been proposed, including genes [41], smoking [42] and psychological stress [43], but the contribution of these putative determinants has yet to be fully elucidated.

It has been suggested that some of the previous research linking obesity to poor health and mortality has failed to take account adequately of physical activity or physical fitness [44] and the relative contributions of fitness and fatness to the development of CVD and its associated risk factors is still debated [45]. Data from this study support an increasing body of work that physical activity is important in terms of being metabolically healthy, independently of adiposity[44]. This supports a move away from a focus primarily on weight loss to reduce CVD and diabetes risk and towards promoting increased physical activity and cardiorespiratory fitness [46] both for the beneficial effects on health it confers independently of weight loss and its role as an important weight management tool.

As discussed above, the factors that we identify explain only a relatively small amount of the risk of having a metabolic abnormality in these very obese women. Future work should focus on identifying other contributory factors. One putative risk factor for poor metabolic health is accumulation of fat in the liver. High liver fat content leads to raised very low density lipoprotein levels and is associated with dysglycaemia[47,48]. The possibility that the women who had a metabolic abnormality in this study had high liver fat content is consistent with the suggestion that these women are impaired in their ability to store fat appropriately in the subcutaneous depot. In addition an association between liver fat and habitual physical activity has been observed, which is consistent with our findings[49]. Other factors worthy of consideration include aspects of diet [50] and duration of or age at onset of obesity[8].

Strengths of this study include the detailed metabolic assessments and the accurate assessment of body fat distribution that were undertaken on each of the participants. The small number of participants in this study, which arose from clinical observations and is intended as a pilot study to inform future work, and the relatively weak measures of physical activity and insulin resistance that were used for pragmatic reasons mean that the strength of reported associations may have been underestimated, but nevertheless the substantial amount of variance in metabolic status that remains unexplained suggests that other factors remain to be identified.

## Conclusion

In summary, our results confirm that individuals exist who are obese but do not appear to suffer the negative metabolic consequences of obesity. Lower levels of intra-abdominal fat, less insulin resistance and higher levels of physical activity are all associated with better metabolic health even in very obese individuals. These findings could be used to guide strategies to identify those obese individuals most at risk of the negative metabolic consequences of obesity. Intra-abdominal fat, insulin resistance and physical activity together account for only around a third of the risk of having a metabolic abnormality in obese women. Future work should aim to identify other contributory factors.

## Competing interests

The authors declare that they have no competing interests.

## Authors' contributions

LH was involved in the study design and was responsible for data collection and analysis and drafting of the manuscript. MP provided statistical advice and contributed to the drafting and revision of the manuscript. MF developed and validated the method for measuring abdominal fat using MRI and commented on drafts of the manuscript. MW was jointly responsible for the idea for the study and its design and has contributed to the development of this manuscript. RT was involved in guiding the data collection methods and analysis for the study and has contributed to the development of this manuscript. NU was jointly responsible for the idea for the study and its design, was involved in data analysis and has contributed to the development of this manuscript. All authors read and approved the final version of the manuscript for publication.

## Pre-publication history

The pre-publication history for this paper can be accessed here:

http://www.biomedcentral.com/1471-2458/10/723/prepub
